# Comparison of nasal intermittent positive pressure ventilation and continuous positive airway pressure on improving bronchopulmonary dysplasia outcomes in preterm infants

**DOI:** 10.3389/fped.2026.1840489

**Published:** 2026-05-22

**Authors:** Yu Dai, Ping Chen, Yumeng Wang

**Affiliations:** 1 Clinical Medical College of Pediatrics, Anhui Second Medical College, Hefei, Anhui Province, China; 2 Department of Pediatrics, The Second Hospital of Anhui Medical University, Hefei, Anhui Province, China; 3 School of Medical Technology, Anhui Second Medical College, Hefei, Anhui Province, China

**Keywords:** bronchopulmonary dysplasia, continuous positive airway pressure, nasal intermittent positive pressure ventilation, preterm infants, respiratory support

## Abstract

**Purpose:**

To compare early nasal intermittent positive pressure ventilation (NIPPV) vs. nasal continuous positive airway pressure (NCPAP) in preterm infants and to evaluate their associations with bronchopulmonary dysplasia (BPD) and respiratory outcomes.

**Methods:**

This retrospective cohort study included 200 preterm infants who received either NIPPV (*n* = 100) or NCPAP (*n* = 100) as initial non-invasive respiratory support within 24 h after birth. Data on respiratory support, arterial blood gases, BPD at 36 weeks postmenstrual age, reintubation, feeding outcomes, other morbidities, and hospitalization duration were compared.

**Results:**

The primary outcome was bronchopulmonary dysplasia (BPD). The incidence of BPD was lower in the NIPPV group (*P* = 0.011). Secondary outcomes included lower rates of invasive ventilation (*P* = 0.010), higher minimum pH (*P* = 0.017), lower maximum partial pressure of carbon dioxide (PaCO_2_) (*P* = 0.011), higher minimum partial pressure of oxygen (PaO_2_) (*P* = 0.037), shorter duration of oxygen therapy (*P* = 0.012), lower reintubation rate within the first week (*P* = 0.010), and shorter hospital stay (*P* = 0.025). Multivariable analysis identified NIPPV as an independent protective factor against BPD (OR = 0.446, *P* = 0.005). No significant differences were observed in other neonatal morbidities or feeding outcomes.

**Conclusion:**

Early NIPPV compared to NCPAP is associated with reduced need for invasive ventilation, improved gas exchange, lower incidence of BPD, and shorter hospitalization in preterm infants without increasing adverse events.

## 1 Introduction

Bronchopulmonary dysplasia (BPD) is a chronic pulmonary condition predominantly affecting premature infants, especially those delivered at less than 32 weeks of gestation. Characterized by abnormal lung development and impaired alveolar growth, BPD remains a major contributor to morbidity and mortality in neonatal intensive care settings. Effective early respiratory support strategies are critical to minimizing BPD risk (1–3).

Non-invasive respiratory support such as nasal continuous positive airway pressure (NCPAP) is widely used to manage preterm infants with respiratory distress syndrome (RDS). NCPAP provides continuous distending pressure to maintain lung volume and reduce work of breathing, decreasing the need for invasive ventilation (4–6). However, NCPAP has limitations, including potential insufficient inspiratory pressure, which may lead to inadequate alveolar recruitment and increased work of breathing. Some infants managed with NCPAP still require intubation and mechanical ventilation, increasing BPD risk (7, 8).

Nasal intermittent positive pressure ventilation (NIPPV) combines NCPAP benefits with additional inspiratory pressure support. By delivering synchronized pressure cycles during inspiration and expiration, NIPPV aims to enhance alveolar recruitment and improve gas exchange more effectively than NCPAP (9, 10). This may reduce the work of breathing and minimize invasive ventilation, thereby mitigating BPD risk. Studies suggest NIPPV offers advantages over NCPAP in respiratory stability and reintubation rates (11, 12).

Despite theoretical advantages of NIPPV, clinical practice varies. Choice between modalities often depends on institutional preferences and clinician experience. Rigorous evaluation of NIPPV compared to NCPAP, particularly in reducing BPD incidence, is needed. This study compares outcomes of NIPPV vs. NCPAP in a cohort of preterm infants.

## 2 Materials and methods

### 2.1 Study design and population

This retrospective cohort study was conducted at the neonatal intensive care unit (NICU) of the Second Hospital of Anhui Medical University in China. The study protocol was reviewed and approved by the Institutional Review Board of the participating center, and informed consent was not obtained owing to the retrospective design, which entailed analysis of anonymized data from pre-existing medical records.

**Figure 1:**
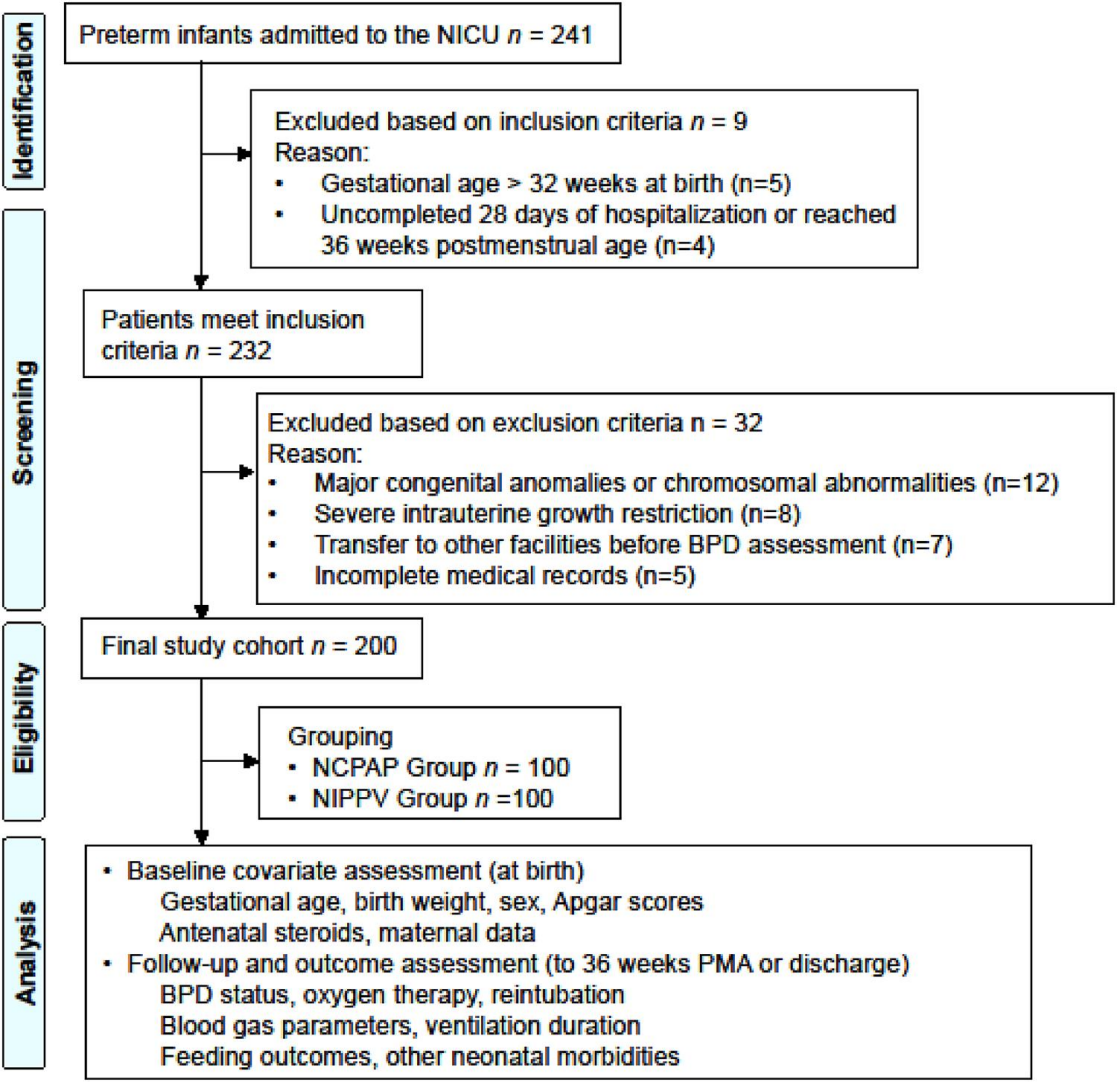
Patient flow diagram.

Preterm infants admitted to the NICU between June 2023 and June 2025 were screened for eligibility. Infants were included if they: (1) gestational age less than 32 weeks at birth, as determined by first-trimester ultrasound or maternal last menstrual period; (2) birth weight < 1,500 g; (3) received either nasal continuous positive airway pressure (NCPAP) or nasal intermittent positive pressure ventilation (NIPPV) as the initial non-invasive respiratory support modality within the first 24 h after birth. Infants were excluded if they met any of the following criteria: (1) major congenital anomalies or chromosomal abnormalities, including congenital heart disease (excluding patent ductus arteriosus and atrial septal defect), congenital diaphragmatic hernia, or tracheoesophageal fistula; (2) severe intrauterine growth restriction, characterized by birth weight < third percentile for gestational age (excluded because these infants often have distinct placental insufficiency-related pathophysiology and poorer respiratory outcomes that may confound the association between respiratory support modality and BPD); or (3) incomplete medical records precluding data collection for key study variables (Figure 1).

**Figure 2:**
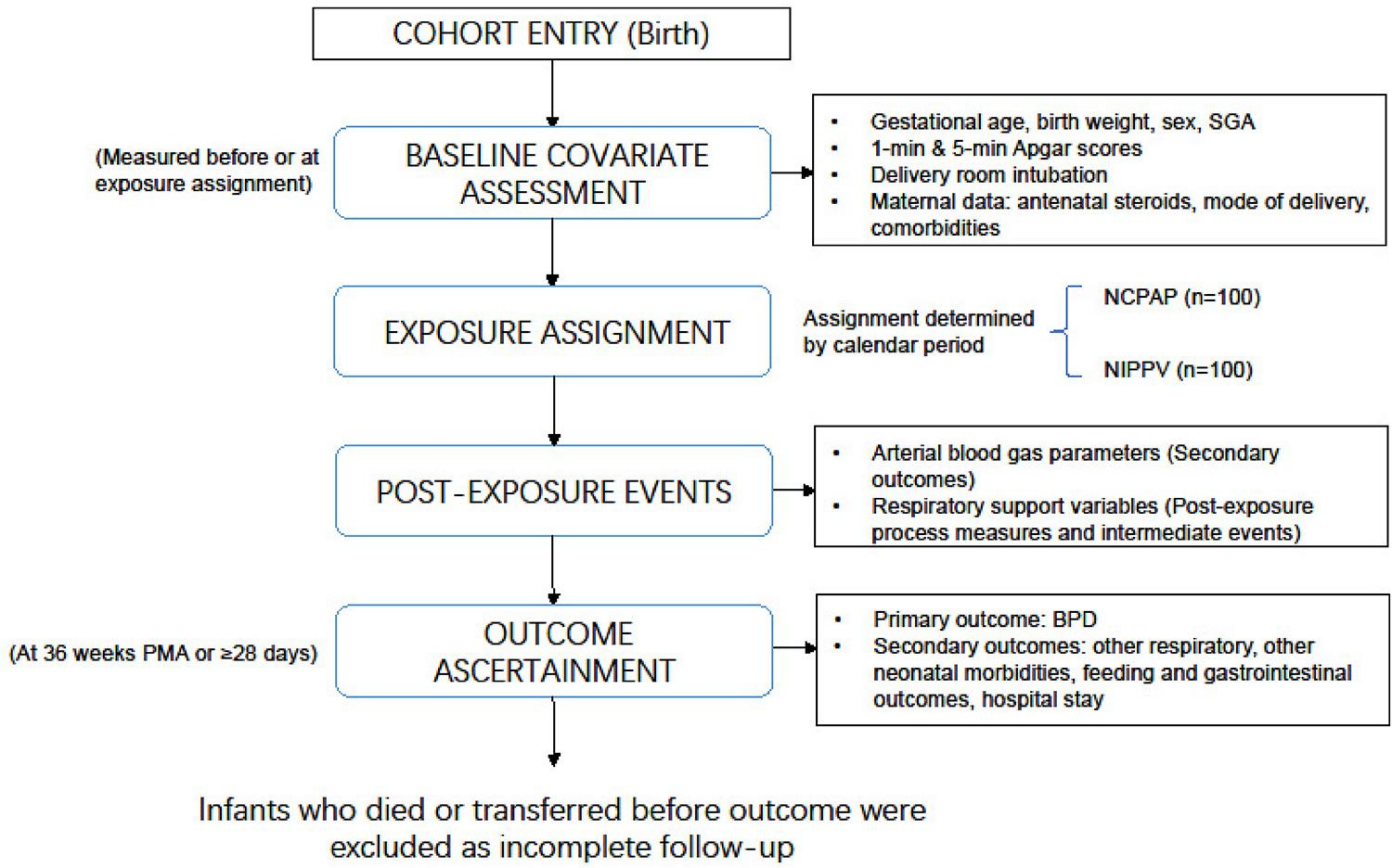
Temporal design diagram of the study.

The assignment to NIPPV vs. NCPAP was based on the calendar period of admission. 100 infants were defined to the NCPAP group and 100 infants to the NIPPV group. The temporal design is illustrated in Figure 2.

### 2.2 Baseline covariates

#### 2.2.1 Maternal data

Maternal data collected included maternal age at delivery, parity (primiparous vs. multiparous), mode of delivery (vaginal delivery vs. cesarean section), administration and completion of antenatal steroids (complete course defined as two intramuscular doses of 12 mg betamethasone given 24 h apart, or incomplete course defined as partial or no administration), presence of hypertensive conditions during pregnancy (chronic hypertension, gestational hypertension, or preeclampsia), gestational diabetes mellitus (diagnosed by oral glucose tolerance testing according to institutional criteria), clinical chorioamnionitis (characterized by maternal temperature > 38.0 °C plus at least two of: maternal or fetal tachycardia, uterine tenderness, or purulent amniotic fluid), and premature rupture of membranes lasting more than 18 h prior to delivery.

#### 2.2.2 Neonatal data

Neonatal data collected included gestational age at birth (determined by first-trimester ultrasound or, when unavailable, by maternal last menstrual period), birth weight measured immediately after delivery using an electronic infant scale (Seca 376, Seca GmbH, Hamburg, Germany), sex (male or female), small for gestational age status, referring to birth weight beneath the 10th percentile for gestational age based on Fenton growth charts, along with 1 min and 5 min Apgar scores as assigned by the attending delivery room team, and need for delivery room intubation (defined as endotracheal intubation performed in the delivery room prior to NICU admission).

### 2.3 Exposure assignment

All infants received respiratory support according to standardized clinical guidelines adapted from the European Consensus Guidelines (13) on the Management of Respiratory Distress Syndrome. During the study period, a practice change occurred in the unit. From June 2023 to June 2024, NCPAP was the routinely preferred initial non-invasive support. In July 2024, synchronized NIPPV became available following equipment updates and staff training; thereafter, NIPPV gradually replaced NCPAP as the preferred initial modality. Consequently, the initial support mode was largely determined by the calendar period of admission rather than by individual disease severity. Infants admitted during the first period (June 2023–June 2024) all received NCPAP as initial support (*n* = 100). During the second period (July 2024–June 2025), NIPPV became the preferred modality; 100 infants who received NIPPV as initial support were included in the NIPPV group. (Infants who received NCPAP during the second period were few and were excluded to maintain a clean comparison. No other changes in neonatal care protocols occurred during the study period, as verified by unit records.

NCPAP was delivered using either the Infant Flow SiPAP system (CareFusion, Yorba Linda, CA, USA) or ventilator-generated CPAP via the Puritan Bennett 980 ventilator (Covidien, Boulder, CO, USA). Initial CPAP settings included positive end-expiratory pressure (PEEP) between 4 and 6 cm H₂O, with fraction of inspired oxygen (FiO_2_) adjusted to maintain oxygen saturation between 90% and 95% as measured by continuous pulse oximetry.

NIPPV was delivered using synchronized or non-synchronized ventilation modes via the same nasal interfaces. Ventilator-generated NIPPV was provided using the Puritan Bennett 980 ventilator (Covidien, Boulder, CO, USA) or the Hamilton-C3 ventilator (Hamilton Medical AG, Bonaduz, Switzerland), both of which are capable of delivering non-invasive positive pressure ventilation to neonatal patients. Initial NIPPV settings included peak inspiratory pressure (PIP) of 14–20 cm H_2_O, PEEP of 4–6 cm H_2_O, ventilation rate of 20–40 breaths per minute, and inspiratory time of 0.3–0.5 s.

Adjustments to ventilator settings were made based on continuous clinical monitoring, including assessment of respiratory effort, chest wall movement, vital signs, and periodic blood gas analyses. Target oxygen saturation range was 90%–95% for all infants, with FiO_2_ adjusted accordingly. Target partial pressure of carbon dioxide (PaCO₂) was maintained between 45 and 55 mmHg, with permissive hypercapnia accepted in the absence of severe acidosis (pH < 7.20).

Criteria for intubation and transition to invasive mechanical ventilation were standardized and included any of the following: (1) persistent apnea defined as apnea lasting >20 s, or accompanied by bradycardia (heart rate < 100 beats per minute) or desaturation (SpO_2_ < 80%) despite loading dose of caffeine citrate (20 mg/kg); (2) severe respiratory acidosis with PaCO_2_ greater than 65 mmHg and pH less than 7.20 on arterial blood gas analysis; (3) hypoxemia with PaO_2_ less than 50 mmHg despite FiO_2_ greater than 0.6; (4) severe respiratory distress unresponsive to non-invasive support; or (5) hemodynamic instability requiring significant cardiovascular support. When intubation was required, infants received surfactant replacement therapy (poractant alfa, 200 mg/kg) via endotracheal tube, with additional doses administered if clinically indicated based on ongoing oxygen requirements.

Reintubation within the first week of life, when required, occurred after the initiation of non-invasive support and was therefore considered an outcome (treatment failure) or an intermediate event on the causal pathway, not a baseline characteristic. In contrast, delivery room intubation was performed before exposure assignment and was used as a baseline covariate to adjust for initial disease severity.

Caffeine citrate therapy was administered routinely to all infants at risk of apnea of prematurity, with a loading dose of 20 mg/kg followed by a maintenance dose of 5–10 mg/kg per day, continued until at least 34 weeks PMA or until resolution of apneic episodes.

Weaning from non-invasive respiratory support followed a standardized clinical protocol. Once the infant demonstrated sustained clinical stability (respiratory rate < 70 breaths per minute, no retractions or grunting, SpO₂ 90%–95% on FiO₂ ≤ 0.30 for ≥ 12 h), pressure support was gradually reduced. For infants on NCPAP, PEEP was decreased by 1 cm H₂O every 12–24 h to a minimum of 4 cm H_2_O before discontinuation. For infants on NIPPV, PIP was first reduced by 2 cm H_2_O increments to a minimum of 12 cm H_2_O, then the backup rate was lowered to 5–10 breaths per minute, after which NIPPV was directly discontinued without transitioning to NCPAP. If clinical deterioration occurred during weaning (increased FiO_2_ requirement ≥ 0.15, recurrent apnea, or respiratory acidosis), support was increased to the previous effective level.

### 2.4 Post-exposure process measures and intermediate events: respiratory support variables measured after exposure

The following variables were collected to characterize the clinical course after exposure assignment. These variables are not primary or secondary outcomes of the study, but rather describe the respiratory support received and early physiological response. They are presented in the Results section for descriptive comparison between groups, but were not included in the multivariable adjustment to avoid overadjustment bias. Respiratory support variables collected included surfactant administration (yes/no and number of doses), caffeine therapy (yes/no), maximum FiO_2_ during the first 72 h of life (recorded as the highest FiO_2_ value documented in the medical record during this period, expressed as a decimal), duration of non-invasive respiratory support (calculated as the total number of days during which the infant received any form of non-invasive positive pressure support, including NCPAP or NIPPV), need for invasive ventilation after initiation of non-invasive support (defined as requirement for endotracheal intubation at any time during the hospitalization, as recorded in respiratory flow sheets), and postnatal corticosteroid use (defined as administration of any systemic corticosteroid, typically dexamethasone or hydrocortisone, for the prevention or treatment of BPD or to facilitate weaning from respiratory support, with dosing and timing recorded from medication administration records).

### 2.5 Outcome measures

#### 2.5.1 Primary outcome: BPD at 36 weeks PMA

The primary outcome of this study was bronchopulmonary dysplasia (BPD), defined according to the consensus definition by Jobe and Bancalari (14) as the need for supplemental oxygen or any form of respiratory support (including NCPAP, NIPPV, or invasive ventilation) at 36 weeks postmenstrual age. Severity of BPD was classified as mild (no oxygen requirement at 36 weeks PMA), moderate (FiO_2_ less than 0.30 at 36 weeks PMA), or severe (FiO_2_ of 0.30 or greater, or need for positive pressure support including NCPAP or ventilation, at 36 weeks PMA). Assessment of BPD status was performed by review of daily respiratory flow sheets and physician progress notes documenting oxygen requirements and respiratory support modalities at 36 weeks PMA.

BPD status was assessed at 36 weeks postmenstrual age or after completing at least 28 days of hospitalization. Infants who died before reaching 36 weeks PMA or completing 28 days of hospitalization or who were transferred to another facility before BPD assessment were excluded from the analysis because BPD status could not be ascertained. These cases represent incomplete follow-up or missing outcome data rather than baseline exclusion criteria. The proportions were low and balanced between groups, which reduces the likelihood of selection bias; however, we acknowledge that exclusion of these infants may introduce bias if death or transfer was related to the exposure and outcome.

#### 2.5.2 Secondary outcomes: arterial blood gas parameters

Arterial blood gas parameters measured during the first week of life were analyzed as secondary outcomes, including the minimum pH value recorded, maximum and minimum PaCO_2_ values (measured in mmHg), and minimum and maximum PaO_2_ values (measured in mmHg). Blood gas analyses were performed using the GEM Premier 3,500 blood gas analyzer (Instrumentation Laboratory, Bedford, MA, USA), which provides measurements of pH, partial pressures of gases, and electrolytes from arterial blood samples. All blood gas measurements were obtained as part of routine clinical care, with sampling frequency determined by the clinical team based on the infant's respiratory status. All arterial blood gas measurements were obtained after the initiation of non-invasive respiratory support (NIPPV or NCPAP), as the first blood gas was typically drawn within 2–4 h after birth following exposure assignment. Therefore, these parameters represent early post-exposure outcomes rather than baseline covariates.

#### 2.5.3 Secondary outcomes: other respiratory outcomes

Secondary respiratory outcomes included oxygen requirement at 28 days of life (defined as any supplemental oxygen use documented at 28 postnatal days), PMA at oxygen discontinuation (calculated as gestational age at birth plus chronological age in weeks when supplemental oxygen was last required), and reintubation within the first week of life (defined as need for endotracheal intubation within seven days after initial extubation or after initiation of non-invasive support).

#### 2.5.4 Secondary outcomes: other neonatal morbidities

Additional secondary outcomes included other neonatal morbidities: patent ductus arteriosus (PDA), severe intraventricular hemorrhage (IVH) (grade ≥ III), retinopathy of prematurity (ROP), late-onset sepsis, pneumothorax/pulmonary air leak, nasal trauma, and pulmonary hemorrhage. PDA was defined as echocardiographically confirmed ductal patency with left-to-right shunting requiring medical (indomethacin or ibuprofen) or surgical treatment; IVH was identified by cranial ultrasound and graded using the Papile classification system (grades I through IV), with severe IVH defined as grade III or IV; ROP was diagnosed by serial ophthalmologic examinations and categorized using the International Classification of Retinopathy of Prematurity, with any stage recorded and treatment-requiring ROP defined as cases receiving laser therapy or anti-vascular endothelial growth factor treatment; late-onset sepsis, defined as culture-proven bacterial or fungal infection occurring after 72 h of life, with organisms identified from blood or cerebrospinal fluid cultures; pneumothorax or pulmonary air leak, diagnosed by chest radiography or transillumination and requiring intervention (needle aspiration or chest tube placement); nasal trauma, defined as any documented injury to the nares or nasal septum including erythema, breakdown, or necrosis related to nasal interfaces; and pulmonary hemorrhage, defined as fresh blood aspirated from the endotracheal tube or visible bleeding into the airways with associated clinical deterioration.

#### 2.5.5 Secondary outcomes: feeding and gastrointestinal outcomes

Feeding and gastrointestinal outcomes were analyzed as secondary outcomes. Feeding and gastrointestinal outcomes assessed included time to achieve full enteral feeding, defined as the postnatal day when the infant first tolerated 120 mL/kg/day of enteral nutrition for at least 3 consecutive days without significant gastric residuals or feeding interruption. Feeding intolerance was characterized by gastric residuals >50% of the previous feed volume, abdominal distension, bilious or bloody gastric aspirates, or emesis leading to temporary cessation or reduction of enteral feeds. Necrotizing enterocolitis (NEC) was diagnosed and staged according to modified Bell's criteria, with stage II or greater (definite NEC) confirmed by the attending neonatologist and documented in the medical record; cases were identified through review of daily progress notes and discharge summaries. Gastrointestinal perforation was defined as radiologically confirmed pneumoperitoneum or surgical evidence of intestinal perforation, with or without associated NEC. Abdominal distension was recorded as a clinical finding documented by the medical team leading to modification of enteral feeding or diagnostic evaluation.

#### 2.5.6 Secondary outcomes: hospitalization and discharge

Duration of hospitalization was calculated as the total number of days from birth to discharge from the NICU or death. Postmenstrual age at discharge was derived as gestational age plus chronological age in weeks at the time of discharge. Weight at discharge was measured using the same electronic infant scale (Seca 376, Seca GmbH, Hamburg, Germany) on the day of discharge. Discharge on oxygen was defined as infants who were discharged home with supplemental oxygen therapy, as documented in discharge orders.

### 2.6 Statistical analysis

Statistical analyses used SPSS 29.0 (IBM Corp., Armonk, NY). Normality was assessed with the Shapiro–Wilk test. Data following a normal distribution are reported as mean ± standard deviation (SD) with two decimal places, whereas non-normally distributed data were intended to be reported as median with interquartile range (IQR), although all continuous variables in the final analysis met normality assumptions. Categorical data are expressed as numbers and percentages. Comparisons between the NIPPV and NCPAP groups were performed using the independent samples t-test for continuous variables and the chi-square test for categorical variables. All statistical tests were two-tailed, and a *P*-value less than 0.05 was considered statistically significant.

Univariable and multivariable logistic regression analyses were conducted to identify independent risk factors associated with bronchopulmonary dysplasia. Variables with *P* < 0.20 in univariable analysis were entered into the multivariable model using a forward stepwise selection procedure, as this threshold is commonly recommended to avoid omitting potentially important confounders. Because variables such as duration of invasive ventilation and maximum FiO_2_ during the first 72 h may lie on the causal pathway between the initial respiratory support modality and BPD (outcome), adjusting for them could introduce overadjustment bias. Therefore, these two variables were excluded from the multivariable analysis. The final multivariable analysis included only baseline covariates measured before or at the time of exposure assignment (gestational age, birth weight, male sex, antenatal steroids), respiratory support modality, and surfactant administration. Surfactant administration was retained because it is a standard clinical covariate not directly on the causal pathway between the two non-invasive modalities and BPD. Results of the multivariable analysis were presented as adjusted odds ratios (OR) with 95% confidence intervals (CI) and corresponding *P*-values.

## 3 Results

### 3.1 Baseline characteristics

241 preterm infants admitted to the NICU between June 2023 and June 2025 were initially screened for eligibility and 232 meet the inclusion criteria. Among these, 200 infants comprised the final study cohort after applying the exclusion criteria. The reasons for exclusion were major congenital anomalies (*n* = 12), severe intrauterine growth restriction (*n* = 8), transfer to other facilities before BPD assessment (*n* = 7), and incomplete medical records (*n* = 5). As shown in Table 1, the two period-defined groups were similar in all measured baseline characteristics, including gestational age, birth weight, sex, Apgar scores, delivery room intubation rate, and proportion of small-for-gestational-age infants (all *P* > 0.05). The absence of baseline differences across the two calendar periods suggests that patient acuity did not systematically differ between the time when NCPAP was the preferred modality and the time when NIPPV became preferred. This supports the validity of comparing outcomes by period as a proxy for exposure assignment.

**Table 1:** Baseline demographic characteristics of the study population.

Variable	NCPAP group (*n* = 100)	NIPPV group (*n* = 100)	t/*χ*^2^	*P*
Gestational age (weeks)	29.34 ± 0.81	29.48 ± 0.74	1.251	0.212
Birth weight (g)	1,173.52 ± 141.27	1,185.84 ± 134.53	0.631	0.528
Male sex, *n* (%)	56 (56%)	57 (57%)	0.020	0.887
Small for gestational age, *n* (%)	21 (21%)	20 (20%)	0.031	0.861
1 min Apgar score	5.59 ± 1.47	5.73 ± 1.42	0.717	0.474
5 min Apgar score	7.42 ± 1.26	7.54 ± 1.21	0.685	0.494
Delivery room intubation, *n* (%)	25 (25%)	22 (22%)	0.250	0.617

NCPAP, Nasal Continuous Positive Airway Pressure; NIPPV, Nasal Intermittent Positive Pressure Ventilation.

### 3.2 Perinatal and maternal characteristics

 Table 2 presents the perinatal and maternal characteristics. The frequency of complete antenatal steroid administration was similar in the NCPAP and NIPPV groups (P = 0.747). No significant differences were found in the rates of cesarean section, multiple gestation, maternal hypertension, maternal diabetes, chorioamnionitis, or premature rupture of membranes (all *P* > 0.05), confirming that the maternal and perinatal backgrounds were comparable between the two cohorts.

**Table 2:** Perinatal and maternal characteristics.

Variable	NCPAP group (*n* = 100)	NIPPV group (*n* = 100)	χ^2^	*P*
Antenatal steroids (complete course), *n* (%)	73 (73%)	75 (75%)	0.104	0.747
Cesarean section, *n* (%)	59 (59%)	60 (60%)	0.021	0.885
Multiple gestation, *n* (%)	23 (23%)	22 (22%)	0.029	0.866
Maternal hypertension, *n* (%)	27 (27%)	26 (26%)	0.026	0.873
Maternal diabetes, *n* (%)	14 (14%)	15 (15%)	0.040	0.841
Chorioamnionitis, *n* (%)	11 (11%)	10 (10%)	0.053	0.818
Premature rupture of membranes (>18 h), *n* (%)	24 (24%)	22 (22%)	0.113	0.737

NCPAP, Nasal Continuous Positive Airway Pressure; NIPPV, Nasal Intermittent Positive Pressure Ventilation.

### 3.3 Respiratory support and surfactant therapy

 Table 3 displays the details of respiratory support and surfactant therapy. While most parameters were similar between groups, including surfactant administration, number of surfactant doses, caffeine therapy, maximum FiO₂ during the first 72 h, duration of non-invasive support, and postnatal corticosteroid use (all *P* > 0.05), a significant difference was found in the need for invasive ventilation. Infants initially managed with NCPAP required invasive ventilation significantly more often than those in the NIPPV group (*P* = 0.010).

**Table 3:** Respiratory support and surfactant therapy.

Variable	NCPAP group (*n* = 100)	NIPPV group (*n* = 100)	t/χ^2^	*P*
Surfactant administration, *n* (%)	68 (68%)	65 (65%)	0.202	0.653
Number of surfactant doses	1.25 ± 0.49	1.20 ± 0.45	0.854	0.394
Caffeine therapy, *n* (%)	92 (92%)	94 (94%)	0.307	0.579
Maximum FiO_2_ during first 72 h (%)	0.42 ± 0.16	0.39 ± 0.14	1.312	0.191
Duration of non-invasive support (days)	17.06 ± 7.24	15.83 ± 6.61	1.258	0.210
Need for invasive ventilation (any), *n* (%)	40 (40%)	23 (23%)	6.697	0.010
Postnatal corticosteroid use, *n* (%)	16 (16%)	11 (11%)	1.070	0.301

NCPAP, Nasal Continuous Positive Airway Pressure; NIPPV, Nasal Intermittent Positive Pressure Ventilation; FiO_2_, Fraction of Inspired Oxygen.

### 3.4 Arterial blood gas and oxygenation parameters

**Figure 3:**
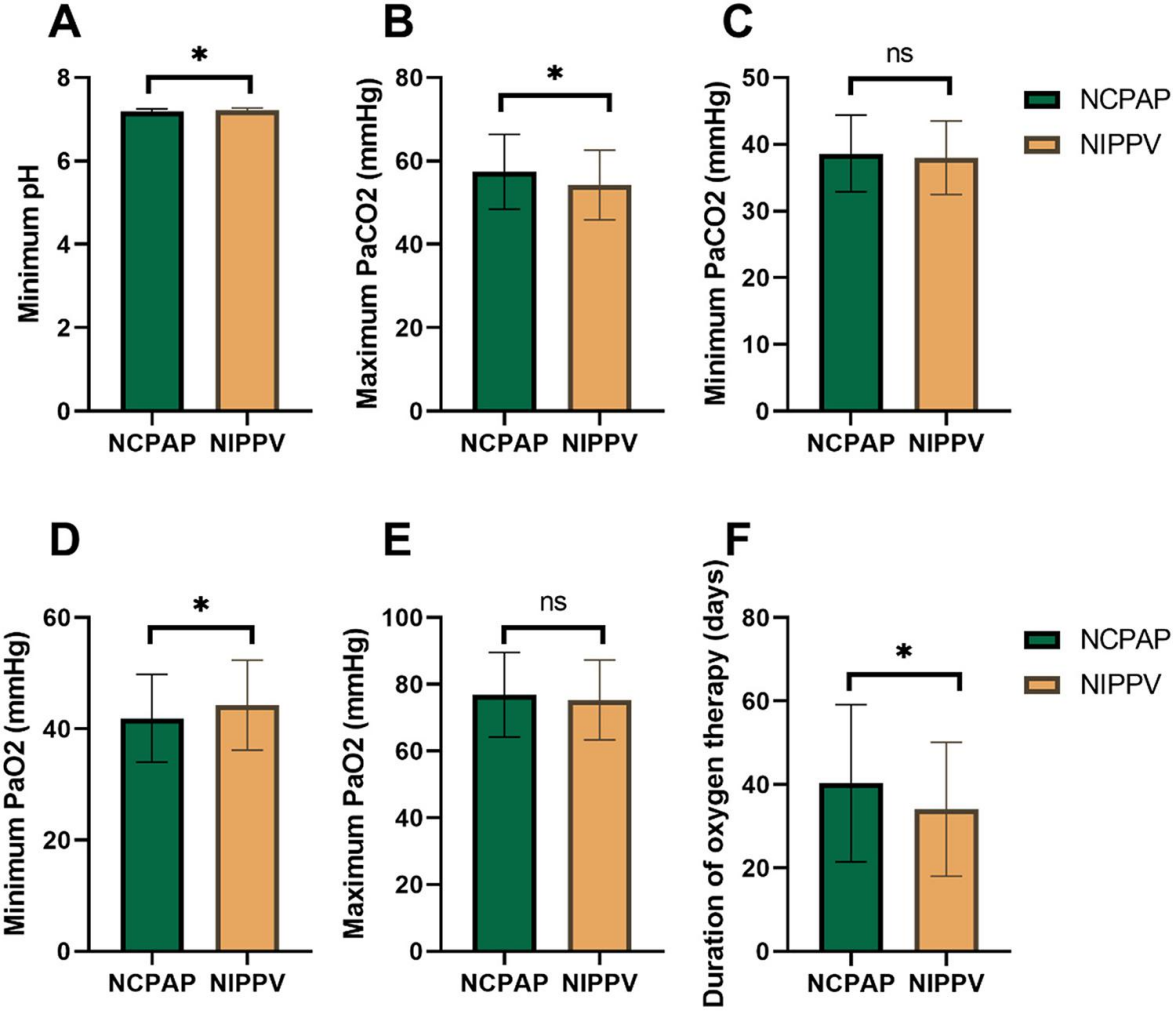
Arterial blood Gas and oxygenation parameters during first week of life. **(A)** Minimum pH; **(B)** Maximum PaCO_2_; **(C)** Minimum PaCO_2_; **(D)** Minimum PaO_2_; **(E)** Maximum PaO_2_; **(F)** Duration of oxygen therapy. ns: no significant difference; *: *P* < 0.05. NCPAP, Nasal Continuous Positive Airway Pressure; NIPPV, Nasal Intermittent Positive Pressure Ventilation; PaCO_2_, Partial Pressure of Carbon Dioxide; PaO_2_, Partial Pressure of Oxygen.

 Figure 3 compares arterial blood gas measurements and oxygenation parameters. Infants in the NIPPV group had a significantly higher minimum pH (7.21 ± 0.06 vs. 7.18 ± 0.07, *P* = 0.017) and a lower maximum PaCO₂ (54.25 ± 8.34 mmHg vs. 57.41 ± 8.96 mmHg, *P* = 0.011) compared with those in the NCPAP group. The minimum PaO₂ was also significantly higher in the NIPPV group (44.26 ± 8.09 mmHg vs. 41.88 ± 7.91 mmHg, *P* = 0.037), and the total duration of oxygen therapy was shorter (34.06 ± 16.03 days vs. 40.34 ± 18.81 days, *P* = 0.012). No differences were found in minimum PaCO₂ or maximum PaO₂ (*P* > 0.05).

### 3.5 Bronchopulmonary dysplasia and respiratory outcomes

The primary outcome, BPD at 36 weeks postmenstrual age, is summarized in Table 4 along with secondary respiratory outcomes. The incidence of bronchopulmonary dysplasia of any grade was lower in the NIPPV group (*P* = 0.011). Additionally, the need for reintubation within the first week of NIPPV group was markedly reduced (*P* = 0.010). The rate of oxygen requirement at 28 days did not differ significantly in groups (*P* = 0.059).

**Table 4:** Bronchopulmonary dysplasia and respiratory outcomes at 36 weeks PMA.

Variable	NCPAP group (*n* = 100)	NIPPV group (*n* = 100)	χ^2^	*P*
BPD (any grade), *n* (%)	56 (56%)	38 (38%)	6.503	0.011
Oxygen requirement at 28 days, *n* (%)	68 (68%)	55 (55%)	3.569	0.059
Reintubation within first week, *n* (%)	34 (34%)	18 (18%)	6.653	0.010

NCPAP, Nasal Continuous Positive Airway Pressure; NIPPV, Nasal Intermittent Positive Pressure Ventilation; BPD, Bronchopulmonary Dysplasia; PMA, Postmenstrual Age.

### 3.6 Feeding and gastrointestinal outcomes

 Table 5 presents feeding and gastrointestinal outcomes. All assessed parameters of two groups, including time to achieve full enteral feeding, feeding intolerance, necrotizing enterocolitis ≥ stage II, gastrointestinal perforation, and abdominal distension, were comparable (*P* > 0.05).

**Table 5:** Feeding and gastrointestinal outcomes.

Variable	NCPAP group (*n* = 100)	NIPPV group (*n* = 100)	t/χ^2^	*P*
Time to full enteral feeding (days)	19.46 ± 8.38	18.62 ± 7.89	0.728	0.467
Feeding intolerance, *n* (%)	26 (26%)	32 (32%)	0.874	0.350
Necrotizing enterocolitis (≥ stage II), *n* (%)	9 (9%)	10 (10%)	0.058	0.809
Gastrointestinal perforation, *n* (%)	3 (3%)	1 (1%)	0.255	0.614
Abdominal distension, *n* (%)	13 (13%)	16 (16%)	0.363	0.547

NCPAP, Nasal Continuous Positive Airway Pressure; NIPPV, Nasal Intermittent Positive Pressure Ventilation.

### 3.7 Other neonatal morbidities and complications

 Table 6 details other neonatal morbidities and complications. Two group's rates of treated patent ductus arteriosus, severe intraventricular hemorrhage, retinopathy of prematurity (any stage or requiring treatment), late-onset sepsis, pneumothorax/pulmonary air leak, nasal trauma, or pulmonary hemorrhage were no significant differences (all *P* > 0.05).

**Table 6:** Other neonatal morbidities and complications.

Variable	NCPAP group (*n* = 100)	NIPPV group (*n* = 100)	χ^2^	*P*
Patent ductus arteriosus (treated), *n* (%)	32 (32%)	30 (30%)	0.094	0.760
IVH (grade ≥ III), *n* (%)	11 (11%)	10 (10%)	0.053	0.818
Retinopathy of prematurity (any stage), *n* (%)	25 (25%)	22 (22%)	0.250	0.617
ROP requiring treatment, *n* (%)	8 (8%)	7 (7%)	0.072	0.788
Late-onset sepsis (culture-proven), *n* (%)	19 (19%)	17 (17%)	0.136	0.713
Pneumothorax/pulmonary air leak, *n* (%)	6 (6%)	5 (5%)	0.096	0.756
Nasal trauma (any grade), *n* (%)	14 (14%)	19 (19%)	0.907	0.341
Pulmonary hemorrhage, *n* (%)	3 (3%)	1 (1%)	0.255	0.614

NCPAP, Nasal Continuous Positive Airway Pressure; NIPPV, Nasal Intermittent Positive Pressure Ventilation; IVH, Intraventricular Hemorrhage; ROP, Retinopathy of Prematurity.

### 3.8 Hospitalization and discharge

 Table 7 shows that NIPPV group's infants had a significantly shorter length of hospital stay (*P* = 0.025). No significant differences were found in postmenstrual age at discharge, weight at discharge, or the proportion discharged on supplemental oxygen (all *P* > 0.05).

**Table 7:** Duration of hospitalization and discharge outcomes.

Variable	NCPAP group (*n* = 100)	NIPPV group (*n* = 100)	t/χ^2^	*P*
Length of hospital stay (days)	65.37 ± 25.51	57.62 ± 23.06	2.255	0.025
PMA at discharge (weeks)	38.78 ± 2.89	38.16 ± 2.71	1.553	0.122
Weight at discharge (g)	2,503.46 ± 445.32	2,568.79 ± 431.47	1.054	0.293
Discharge on oxygen, *n* (%)	13 (13%)	8 (8%)	1.330	0.249

NCPAP, Nasal Continuous Positive Airway Pressure; NIPPV, Nasal Intermittent Positive Pressure Ventilation; PMA, Postmenstrual Age.

### 3.9 Multivariate analysis

 Table 8 presents multivariable logistic regression analysis for factors of bronchopulmonary dysplasia. After adjusting for potential confounders, NIPPV was identified as an independent protective factor against BPD compared with NCPAP (*P* = 0.005). Lower gestational age (*P* < 0.001) and higher birth weight (*P* = 0.001) were protective. Male sex, antenatal steroids, and surfactant administration were not significantly associated (all *P* > 0.05).

**Table 8:** Multivariable logistic regression analysis for factors associated with bronchopulmonary dysplasia.

Variable	Adjusted OR	95% CI	*P*
Gestational age (per 1-week increase)	0.523	0.371–0.738	<0.001
Birth weight (per 100-g increase)	0.691	0.565–0.845	0.001
Male sex	1.584	0.841–2.984	0.154
Antenatal steroids (complete course)	0.619	0.350–1.095	0.099
NIPPV versus NCPAP	0.446	0.254–0.782	0.005
Surfactant administration	1.635	0.881–3.035	0.119

NCPAP, Nasal Continuous Positive Airway Pressure; NIPPV, Nasal Intermittent Positive Pressure Ventilation; OR, Odds Ratio; CI, Confidence Interval; FiO2, Fraction of Inspired Oxygen.

## 4 Discussion

BPD remains a complication in preterm infants, leading to neurodevelopmental impairment. Early respiratory support strategies play a crucial role in mitigating the risk of BPD. This study aimed to compare the effectiveness of NIPPV and NCPAP in improving respiratory outcomes and reducing the incidence of BPD. The findings highlight several key differences between the two modes of respiratory support. Infants initially managed with NCPAP required invasive ventilation more frequently than those in the NIPPV group. Infants in the NIPPV group exhibited improved arterial blood gas parameters compared to those in the NCPAP group, including higher minimum pH, lower maximum PaCO₂, higher minimum PaO₂, and shorter duration of oxygen therapy. The incidence of BPD was lower in the NIPPV group. Additionally, infants in the NIPPV group had a shorter length of hospital stay compared to those in the NCPAP group. No significant differences were observed in complications such as necrotizing enterocolitis, pneumothorax, pulmonary hemorrhage, or nasal trauma between the two groups.

While the observed differences in blood gas parameters between groups were statistically significant, their absolute magnitudes—approximately 0.03 units for pH, 3 mmHg for PaCO₂, and 2 mmHg for PaO₂—are modest. However, even small improvements in acid-base balance and gas exchange may be clinically relevant in extremely preterm infants with limited pulmonary reserve, as they may reduce the cumulative exposure to hypoxemia and hypercapnia. Furthermore, these differences occurred alongside more substantive clinical outcomes, including lower rates of invasive ventilation and BPD, supporting the overall benefit of NIPPV.

Our findings align with previous studies demonstrating advantages of NIPPV over NCPAP in preterm infants. A recent Cochrane review by Lemyre et al. (11) concluded that early NIPPV reduces the risk of respiratory failure and need for intubation compared to NCPAP, consistent with our observation of lower invasive ventilation rates.

The mechanism behind the reduced need for invasive ventilation could be related to the ability of NIPPV to deliver higher pressures during both inspiration and expiration, thereby maintaining alveolar patency more effectively than NCPAP (15, 16). This enhanced pressure support can prevent atelectasis and promote better gas exchange, reducing the likelihood of respiratory failure requiring invasive intervention (17, 18). The improved oxygenation and ventilation in the NIPPV group could be attributed to its ability to generate higher mean airway pressures and more effective clearance of CO₂, leading to better overall lung function (19–21). The reduction in BPD rates could be linked to the improved respiratory support provided by NIPPV, which helps maintain alveolar stability and reduces the need for invasive ventilation. Invasive ventilation is associated with various complications, including barotrauma and volutrauma, which can exacerbate lung injury and increase the risk of BPD. By minimizing the need for invasive ventilation, NIPPV may reduce these adverse effects, thereby lowering the incidence of BPD. Furthermore, the shorter duration of oxygen therapy in the NIPPV group may contribute to reduced oxidative stress on the developing lungs, further protecting against BPD (22–24). The shorter hospital stay could be attributed to the reduced need for invasive ventilation and shorter duration of oxygen therapy, both of which are major contributors to prolonged NICU stays. The improved respiratory status of infants in the NIPPV group may lead to faster achievement of full enteral feeding and fewer complications, further accelerating the discharge process (25–27). Several mechanisms may explain the advantages of NIPPV over NCPAP. NIPPV delivers synchronized pressure cycles during both inspiration and expiration, which can enhance lung volume recruitment and prevent alveolar collapse. This synchronization ensures more consistent and effective ventilation, promoting better gas exchange and reducing the work of breathing. NIPPV can generate higher mean airway pressures, which help maintain adequate lung volumes and reduce the risk of atelectasis. The intermittent nature of NIPPV allows for brief periods of spontaneous breathing, which can stimulate diaphragmatic activity and improve lung compliance. These mechanisms collectively contribute to improved respiratory function and reduced risk of BPD (28–30).

This study has several strengths that make it worthy of reporting. The relatively large cohort of 200 infants enrolled within a two-year period minimizes temporal biases related to changes in clinical practice, providing evidence that is more reflective of current neonatal care. Baseline characteristics were well balanced between groups, reducing selection bias. Comprehensive data collection allowed adjustment for multiple potential confounders in multivariable analysis. The study assessed a wide range of outcomes, including respiratory, feeding, gastrointestinal, and other neonatal morbidities, providing a holistic evaluation of both efficacy and safety of NIPPV.

This study has limitations that warrant consideration. Firstly, the retrospective design introduces potential biases. Prospective randomized controlled trials are needed to confirm these findings. Second, although we used a *P* < 0.20 threshold for variable selection in multivariable analysis, the possibility of residual confounding from unmeasured or excluded variables cannot be completely excluded. The protective effect of NIPPV against BPD remained significant after adjustment for major confounders. Future investigations should seek to overcome these shortcomings through rigorous methodological designs and larger sample sizes. Third, although a standardized weaning protocol was followed, adherence may have varied among attending clinicians, potentially introducing unmeasured bias. This limitation is inherent to retrospective studies. Fourth, the exclusion of infants with severe IUGR limits generalizability to this population. Fifth, the requirement for survival to 36 weeks PMA or completion of 28 days of hospitalization for BPD assessment may introduce selection bias if differential mortality or transfer occurred between the two groups. Although the proportions of infants who died or were transferred before outcome ascertainment were low (≤4% in either group) and similar between groups, we cannot entirely rule out the possibility of selection bias. However, the low event rates suggest that any such bias, if present, is likely small and unlikely to alter the main conclusions. Sixth, although the assignment to NIPPV vs. NCPAP was primarily driven by calendar period rather than clinical judgment, we cannot exclude the possibility of unmeasured temporal confounders. However, no other major protocol changes occurred during the study period, and baseline characteristics were stable across the two periods, which mitigates this concern. Nonetheless, a randomized trial would provide stronger evidence. Additionally, exploring the long-term outcomes of infants treated with NIPPV vs. NCPAP would provide valuable insights into the sustained benefits of early respiratory support strategies. While subgroup analyses by gestational age or other potential effect modifiers were not performed in the current study, future adequately powered studies may explore whether the treatment effect of NIPPV differs across gestational age strata or other clinical subgroups. Incorporating patient-reported outcomes and quality-of-life assessments could further improve insight into the effects of these interventions on infant well-being.

## 5 Conclusion

The primary finding of this study is that early NIPPV significantly reduced the incidence of BPD compared with NCPAP in preterm infants. NIPPV was also associated with reduced need for invasive ventilation, improved gas exchange, shorter oxygen therapy, lower reintubation rate, and shorter hospital stay, without increasing adverse events. These findings suggest that NIPPV may be a beneficial alternative to NCPAP for early respiratory support in preterm infants.

## Data Availability

The raw data supporting the conclusions of this article will be made available by the authors, without undue reservation.
